# Self-Assembling Peptide RADA16 Nanofiber Scaffold Hydrogel-Wrapped Concentrated Growth Factors in Osteogenesis of MC3T3

**DOI:** 10.3390/jfb14050260

**Published:** 2023-05-08

**Authors:** Renjie Yang, Jiali Chen, Dingjie Wang, Yichen Xu, Guomin Ou

**Affiliations:** 1 State Key Laboratory of Oral Diseases & National Clinical Research Center for Oral Diseases, Eastern Clinic, West China Hospital of Stomatology, Sichuan University, Chengdu 610051, China; 2 State Key Laboratory of Oral Diseases & National Clinical Research Center for Oral Diseases, Department of Oral and Maxillofacial Surgery, West China Hospital of Stomatology, Sichuan University, Chengdu 610041, China; 3 State Key Laboratory of Oral Diseases & National Clinical Research Center for Oral Diseases, Department of Oral Implantology, West China Hospital of Stomatology, Sichuan University, Chengdu 610041, China; 4 State Key Laboratory of Oral Diseases & National Clinical Research Center for Oral Diseases, Department of Oral Prosthodontics, West China Hospital of Stomatology, Sichuan University, Chengdu 610041, China

**Keywords:** alveolar bone cleft, alveolar bone grafting, RADA16, CGFs, osteogenesis

## Abstract

Concentrated growth factors (CGFs) are widely used in surgery with bone grafting, but the release of growth factors from CGFs is rapid. RADA16, a self-assembling peptide, can form a scaffold that is similar to the extracellular matrix. Based on the properties of RADA16 and CGF, we hypothesized that the RADA16 nanofiber scaffold hydrogel could enhance the function of CGFs and that the RADA16 nanofiber scaffold hydrogel-wrapped CGFs (RADA16-CGFs) would perform a good osteoinductive function. This study aimed to investigate the osteoinductive function of RADA16-CGFs. Scanning electron microscopy, rheometry, and ELISA were performed, and MC3T3-E1 cells were used to test cell adhesion, cytotoxicity, and mineralization after administration with RADA16-CGFs. We found that RADA16 endowed with the sustained release of growth factors from CGFs, which can help maximize the function of CGFs in osteoinduction. The application of the atoxic RADA16 nanofiber scaffold hydrogel with CGFs can be a new therapeutic strategy for the treatment of alveolar bone loss and other problems that require bone regeneration.

## 1. Introduction

The alveolar cleft is one common congenital craniofacial defect in patients with cleft lip and cleft palate and is often accompanied by tooth agenesis. Alveolar bone grafting is commonly performed to reconstruct the alveolar ridge, stabilize the maxillary arch, and guarantee implant treatment [1]. Iliac cancellous bone grafting is the gold standard treatment for alveolar clefts [2], providing osteoinductive (recruitment of host mesenchymal stem cells from the surrounding tissue) and osteoconductive (scaffolding for cells to climb in and vascularization) [3]. This process is facilitated by the presence of growth factors within the autogenous bone material.

For some patients, due to the lack of donor site morbidity and large volume, allogenic bone and substitute bone could be potential alternative choices [4]. Allogenic bone and substitute bone grafts avoid second surgical sites to simplify the operation and relieve pain [5]. However, compared with autogenous bone grafts, these materials lack osteogenic and osteoinductive properties. Thus, we expect to design a complex bone substitute to provide both scaffold and bioactivity to cover this shortage [6].

Concentrated growth factors (CGFs) are the platelet-rich fibrin (PRF) derivatives that have been applied in the clinic [7], which can release Transforming Growth Factor-β (TGF-β), Vascular Endothelial Growth Factor (VEGF), Platelet-Derived Growth Factor (PDGF), Bone Morphogenetic Protein 2 (BMP2) and Insulin-like Growth Factor (IGF-1) [8,9]. These growth factors can increase proliferation and extracellular matrix mineralization [10]. Studies have shown the osteogenic effect of CGFs in vivo [11,12,13,14] and in vitro [12,15,16]. However, the release of growth factors from CGFs is rapid, while bone formation is slow and complex [16]. To match the process of bone formation, extending the releasing process of the growth factors of CGFs becomes important. Studies have shown that CGF-wrapped scaffolds can help extend the releasing process [17], and different hydrogels, such as chitosan and sodium alginate, have been tested [18].

Self-assembling peptides (SAPs) are oligopeptides that can spontaneously self-assemble into nanostructures under appropriate conditions [19,20]. A series of SAPs have been synthesized, and RADA16 became distinctive because its structure is exceedingly orderly, and its self-assembling can be controlled in a neutral pH solution or a physiological saline solution. RADA16 can form a stable scaffold structure similar to the extracellular matrix (ECM), with excellent biocompatibility and low immunogenicity [21,22]. RADA16 nanofiber scaffold hydrogel can help in cell adhesion and proliferation and provide a suitable microenvironment for cell differentiation [23]. Studies have shown that RADA16 nanofiber scaffold hydrogel could promote osteogenesis and control the release of functional factors when combined with the application of growth factors, such as Bone Morphogenetic Protein-2 (BMP-2) [22,24,25,26].

Based on the properties of RADA16 and CGF, we hypothesized that the RADA16 nanofiber scaffold hydrogel could enhance the function of CGFs and that the RADA16 nanofiber scaffold hydrogel-wrapped CGFs (RADA16-CGFs) would perform osteogenic and osteoinductive functions. Therefore, this study aimed to investigate the osteogenesis function of RADA16-CGFs in vitro to provide a new strategy for the clinic.

## 2. Materials and Methods

### 2.1. Blood Sample Centrifugation and CGF Preparation

**Figure 1:**
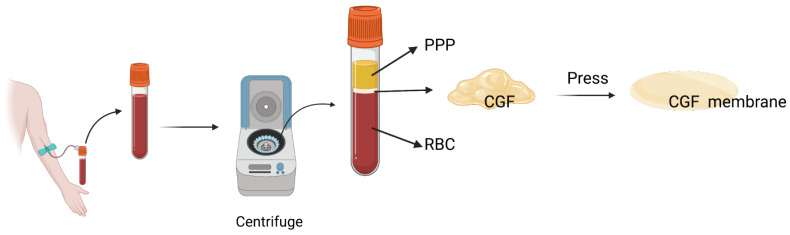
CGF preparation. Blood was drawn from the arm vein, and the tubes were centrifuged under the CGF preparation procedure. Three blood fractions were found at the end of the process. The middle fraction was extracted as the CGF composition and squashed to form the CGF membrane.

Vein blood samples were obtained from 6 healthy volunteers who were nonsmokers from the West China College of Stomatology, Sichuan University. Sample collection was performed with the approval of the Ethics Committee of West China Hospital of Stomatology, Sichuan University (WCHSIRB-D-2021-534). All potential participants signed an informed consent form prior to sample collection and were informed of the potential benefits and risks of participating. The CGFs were produced as follows: 9 mL of blood was drawn from the arm vein in 10 mL blood collection tubes without an anticoagulant solution. The tubes were immediately centrifuged in a special machine (Medifuge MF200, Silfradent Srl, Santa Sofia, Italy) under the CGF preparation procedure. At the end of the process, there were three blood fractions. The middle fraction was extracted as the CGF composition and squashed to form the CGF membrane (Figure 1).

### 2.2. RADA16 Synthesis

**Figure 2:**
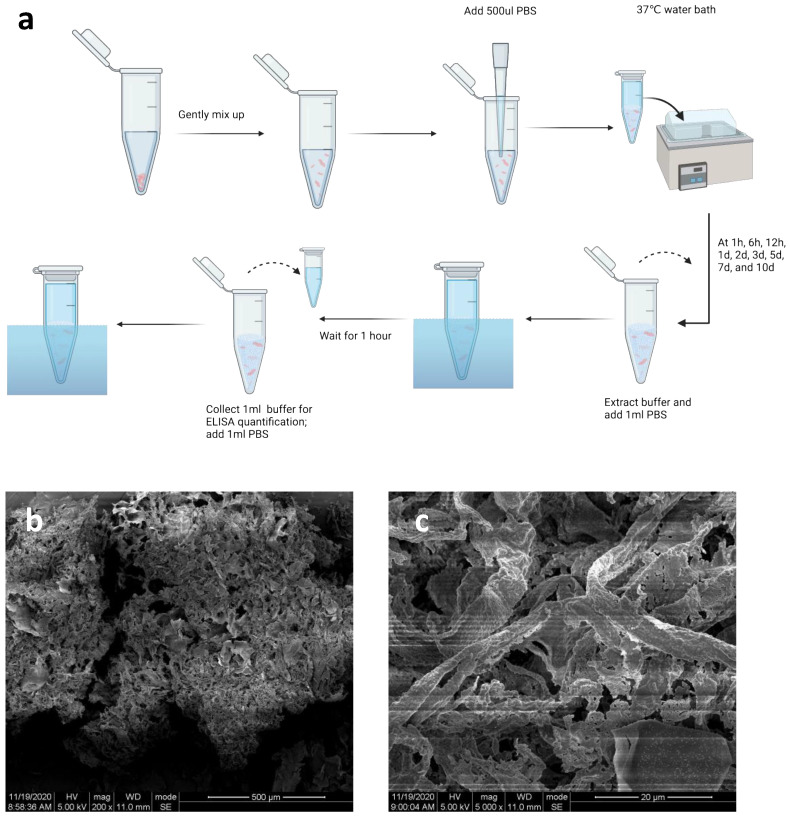
Properties of CGFs. (**a**) Sample preparation for ELISA quantification. Shredded CGF membrane and 2% *w*/*v* RADA16 solution (500 µL) were softly mixed in a 1.5 mL EP tub. Then, 500 μL PBS was added to the tube and put into a 37 °C water bath. The buffer was extracted at 1 h, 6 h, 12 h, 1 d, 2 d, 3 d, 5 d, 7 d, and 10 d, and another 1 mL PBS was added into the tubes for 1 h, and extracted for ELISA quantification. (**b**,**c**) SEM image of CGFs. Bars, 500 μm and 20 μm, respectively.

The RADA16 applied in this study is L-RADA16, of which the peptide sequence is AcN-RADARADAR ADARADA-CONH2 (molecular weight: 1671.76). The RADA16 peptide was custom-synthesized commercially using solid-phase peptide synthesis (Hangzhou Jiatai Co., Ltd., Hangzhou, China). The C-terminus and N-terminus of this peptide were amidated and acetylated, respectively [20]. The peptide was purified using high-performance liquid chromatography and characterized using mass spectroscopy. Samples were dissolved in PBS (pH 7.4) at a concentration of 20.0 mg/mL (2% *w*/*v* RADA16) and stored at 4 °C in a refrigerator for further study (Figure 2a).

### 2.3. RADA16-CGF Fabrication

Shredded CGF membrane and 2% *w*/*v* RADA16 solution (500 µL) were softly mixed in a 1.5 mL EP tube and prepared for further study.

### 2.4. Scanning Electron Microscopy (SEM) Assay

To observe the CGFs, the CGF membrane was dried for 12 h in a lyophilizer before the SEM assay. To observe the RADA16 nanofiber scaffold hydrogel, the peptide solution (2% *w*/*v* RADA16) was mixed with PBS (pH 7.4) at a volume ratio of 1:1 and stored in a 1.5 mL EP tube (1% *w*/*v* RADA16) with sonication for 30 min to decrease viscidity. The peptide solution sample was added to 400 mL PBS and stored at 37 °C overnight. The hydrogel was then dried for 12 h in a lyophilizer. The samples were observed under a scanning electron microscope (Hitachi S-3400N; Hitachi, Ltd., Tokyo, Japan).

### 2.5. Rheometry

Rheology assays were carried out using a 1° stainless steel cone-controlled rheometer with a diameter of 20 mm (Thermo Fisher Scientific, Waltham, MA, USA). Samples were dissolved in PBS (pH 7.4) at a concentration of 10.0 mg/mL the peptide solution (2% *w*/*v* RADA16) was mixed with PBS (pH 7.4) at a volume ratio of 1:1 and stored at 4 °C in a refrigerator overnight, and 150–200 μL of samples were used for analysis at 25 °C.

### 2.6. ELISA Quantification for Growth Factors Released from RADA16-CGFs

One milliliter of PBS was added to the RADA16-CGF tube. The buffer was extracted at 1 h, 6 h, 12 h, 1 d, 2 d, 3 d, 5 d, 7 d, and 10 d, and another 1 mL PBS was added into the tubes for 1 h, and extracted for ELISA quantification. When all samples were collected, quantifications of platelet-derived growth factor-BB (PDGF-BB) and vascular endothelial growth factor (VEGF) were performed using commercially available ELISA kits (Beyotime, Biotech Co., Ltd., Nanjing, China).

### 2.7. Cells

The MC3T3-E1 cells (cat. # CRL-2593) were used in this study, which was purchased from American Type Culture Collection (ATCC) (Manassas, VA, USA). MC3T3-E1 cell cultures were maintained in minimum essential medium (MEM, Gibco; Thermo Fisher Scientific, Inc.) supplemented with 10% fetal bovine serum (FBS, Gibco; Thermo Fisher Scientific, Inc.), and 1% (*v*/*v*) penicillin/streptomycin (Gibco; Thermo Fisher Scientific, Inc.) at 37 °C in a humidified CO_2_ incubator. Cells at approximately 80% confluence were passaged by trypsin digestion and expanded through two passages before being used for the study.

### 2.8. Cell Culture Medium Preparation

CGFe (CGF extracts) preparation: CGFs after lyophilization overnight were pulverized and immersed in 45 mL DMEM for incubation at 4 °C for 24 h. CGFe was collected after centrifugation and bacteriological filtration.

CGF-conditioned medium (CCM) preparation: CCM was completed with CGFe supplemented with 10% fetal bovine serum and 1% antibiotic.

Osteogenic medium (OM) preparation: 0.5 g vitamin C (Vc) was dissolved in 10 mL PBS (Solution I). A total of 3.0611 g C_3_H_7_Na_2_O_6_P_5_ (H_2_O) was dissolved in 9.1 mL PBS (Solution II). One milligram of hexadecadrol was dissolved in 1 mL ethanol, and 200 µL of this solution was added into 10 mL α-MEM (Solution III). Then, 50 mL of medium was mixed with 50 µL Solution I, 500 µL Solution II, and 500 µL Solution III.

CGF-containing Osteogenic medium (CCOM) preparation: CGFe was mixed with 10% fetal bovine serum, 1% antibiotic, 50 µL Solution I, 500 µL Solution II, and 500 µL Solution III.

### 2.9. Cell Adhesion Experiment

24 well plates were prepared. A total of 3 × 10^4^ MC3T3-E1 cells were seeded in each well. Four groups were designed: (1) Control group: 1 mL α-MEM was added into the well; (2) CGFs group: 1 mL Solution IV was added into the well; (3) RADA16 group: 200 µL 1% *w*/*v* RADA16 was added to the well and stored under 37 °C, 1 mL α-MEM was added and replaced once 15 min until the pH of the medium was close to 7.4, and stored in the incubator overnight before adding the cells; (4) RADA16-CGFs group, the fabrication process was similar to RADA16 group, but change the final time of the addition of 1 mL α-MEM to 1 mL Solution IV. After cell seeding, the cells of each well were observed at 10 min, 30 min, 60 min, and 90 min after incubation. The number of adhesive cells was measured.

### 2.10. Cytotoxicity Experiment

MC3T3-E1 cells were seeded in a 96-well tissue culture plate at a density of 3000 cells per well. The design of the groups was the same as in the cell adhesion experiment, but the total medium was changed to 200 µL per well with the same ratio of different solutions as before. On days 1, 3, 5, and 7, the Cell Counting Kit-8 (CCK-8) assay was applied to determine the overall proliferation cytotoxicity. The absorbance (optical density (OD) value at 450 nm) was read by a microplate reader (Thermo Fisher Scientific, Inc.). To avoid the interference of RADA16, the medium was transferred to a new 96-well plate for measurement.

### 2.11. Alkaline Phosphatase (ALP) Staining

MC3T3-E1 cells were seeded in a 24-well tissue culture plate at a density of 3 × 10^4^ cells per well with the corresponding reagents (1 mL). Five groups were designed: (1) Negative control with α-MEM; (2) Positive control with OM; (3) CGF-OM group; (4) RADA16-OM group; and (5) RADA16-CGFs-OM group. ALP staining (Beyotime, Biotech Co., Ltd., Nanjing, China) was performed after 7 d of incubation.

### 2.12. Alizarin Red S Staining

To identify the mineralization nodules, Alizarin Red S (Solarbio, Beijing, China) staining was performed after the MC3T3-E1 cells were seeded at a density of 3 × 10^4^ cells per well and grew for 21 days in five groups. After gently rinsing with ddH2o, the cells were stained in a solution of 2% ARS at pH 4.1 for 20 min and then washed with ddH_2_O. The samples were air-dried, and images were captured under a light microscope (magnification, ×6). Additionally, the bound dye was dissolved with 10% cetylpyridinium chloride, and the ARS in the samples was quantified by measuring the absorbance at 562 nm.

### 2.13. Alp Gene Expression

For the detection of *Alp* genes (ALP, F: CAGTTCGTATTCCACATCAGTTC R: CAAGGACATCGCATATCAGCT; GAPDH, F: AAGAAGGTGGTGAAGCAGG R: GAAGGTGGAAGAGTGGGAGT), MC3T3-E1 cells were plated at a density of 1 × 10^4^ cells per well in separate 6-well plates in different media of five groups. Total RNA from all groups was extracted using TRIzol reagent after 14 d of culture and analyzed by reverse transcription-quantitative (RT-q) PCR.

### 2.14. Statistical Analysis

All analyses were performed using SPSS 25 software (IBM Corp., Armonk, NY, USA). All experiments were performed in at least three independent repeats. All data are shown as the mean and standard deviation (SD) and were analyzed using one-way ANOVA or a nonparametric test followed by the least significant difference post hoc test. The levels of significance were set at * *p* < 0.05, ** *p* < 0.01, and *** *p* < 0.001 (as indicated in the figures and legends with the corresponding symbols).

## 3. Results

### 3.1. Characterization of CGFs

After blood sample centrifugation, three blood fractions, including the fractions of plate-poor plasma, CGFs, and erythrocytes, were found in the tubes. The middle CGF fraction was extracted. The SEM images demonstrated the architecture and morphology of the CGFs (Figure 2b,c).

### 3.2. Characterization of RADA16

**Figure 3:**
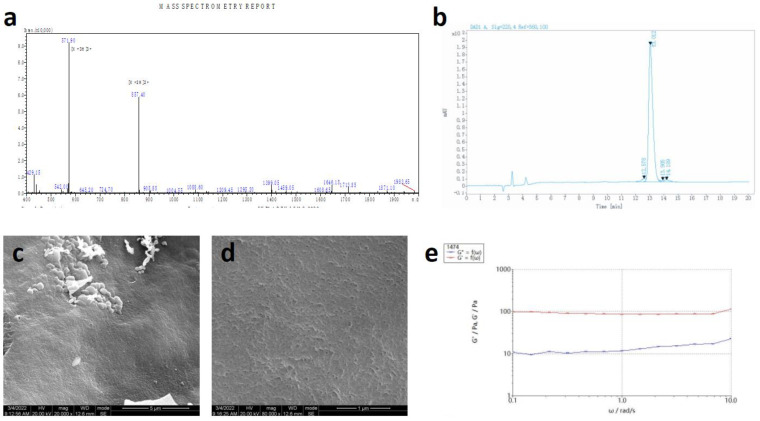
Properties of RADA16. (**a**) Mass spectroscopy of the synthetic peptide. (**b**) High-performance liquid chromatography of synthetic peptides. (**c**,**d**) SEM images of RADA16 (1%). Bars, 5 μm and 1 μm, respectively. (**e**) Rheometry performance of RADA16 (1%).

RADA16 peptide sequences were confirmed by mass spectroscopy and purified by high-performance liquid chromatography (HPLC), and the final purity was 95.22% (Figure 3a,b). SEM images demonstrated the nanostructure of 1% *w*/*v* RADA16 the nanofiber scaffold hydrogel in PBS, which showed that RADA16 peptides were able to self-assemble into nanofibers at 25 °C. These nanofibers were about 20 nm in diameter and formed pores of about 200 nm (Figure 3c,d). The rheological test of 1% *w*/*v* RADA16 nanofiber scaffold hydrogel showed the storage modulus G’ was always greater than the loss modulus G″, and the frequency G′ was always over zero, in 0.1–10 rad/s, which confirmed that saline solution such as PBS can induce RADA16 to self-assemble into nanofiber hydrogel (Figure 3e).

### 3.3. Growth Factor Releasing Process of RADA16-CGF

**Figure 4:**
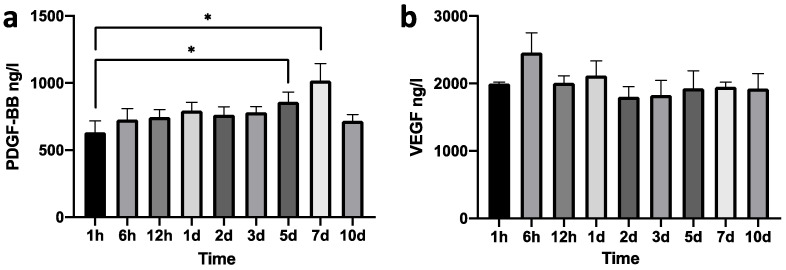
Growth factors released from RADA-CGFs. (**a**) Concentration of PDGF-BB at each time point. *, *p* < 0.05. (**b**) Concentration of VEGF at each time point.

The growth factors releasing CGFs are rapid [27], and the application of RADA16 nanofiber scaffold hydrogel aims to decrease the release. The dynamics of PDGF-BB and VEGF release of RADA16-CGFs are shown in Figure 4a,b. The concentrations of PDGF-BB released at 5 d and 7 d were significantly different from the release at 1 h, while the releases at other time points were not different from the release at 1 h. No difference was found between any time points of the VEGF release. Thus, the releasing process of RADA16-CGFs could be stable and much slower than that of pure CGFs [27].

### 3.4. Cell Proliferation of MC3T3 with RADA16-CGF

**Figure 5:**
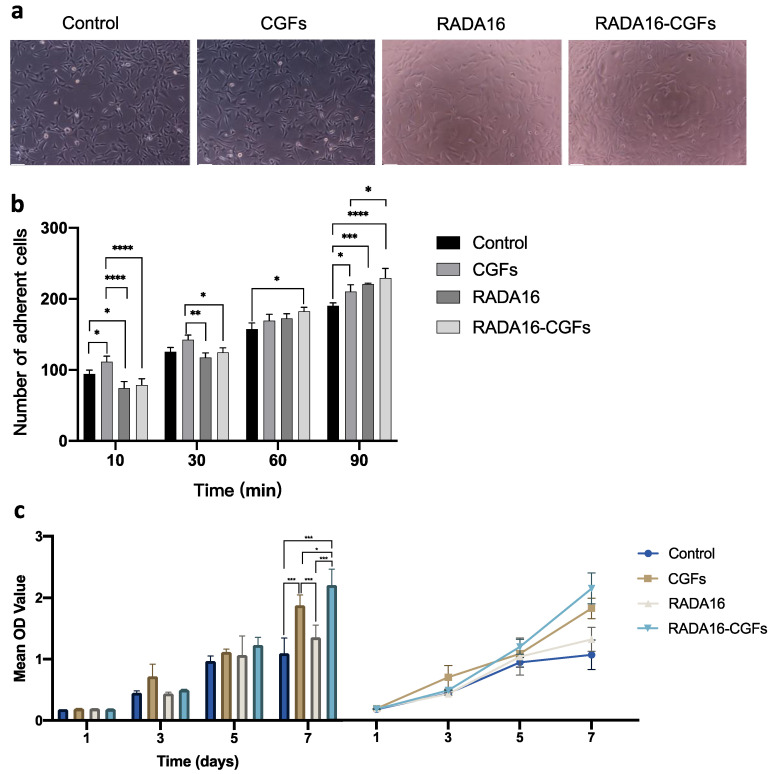
Cell proliferation of MC3T3 with RADA16-CGF. (**a**) MC3T3 cell morphology co-cultured with different composites after 3 days. Scale bars, 100 μm. (**b**) Comparison of the number of adherent cells between the control, CGFs, RADA16, and RADA16-CGF groups at different timepoints. *, *p* < 0.05; **, *p* < 0.005; ***, *p* < 0.001; ****, *p* < 0.0001. (**c**) Comparison and tendency chart of the CCK-8 assays for the proliferation of MC3T3 cells co-cultured with different composites. *, *p* < 0.05; ***, *p* < 0.001.

Cell culturing with CGFs, RADA16, and RADA16-CGFs did not affect the cell morphology, and the RADA16-CGF group had better cell proliferation than the other three groups after 72 h (Figure 5a). By measuring the cell adhesion, we found that in the early stage (10–30 min), CGFs could help the cell adhesion, while the RADA16 and RADA16-CGFs could decrease the cell adhesion; but later (90 min), all CGFs, RADA16, and RADA16-CGFs groups had better cell adhesion than the control group, and RADA16-CGFs had the best effect, which further indicated the function of the RADA16 nanofiber scaffold hydrogel (Figure 5b). By analyzing the results of CCK-8 assays, there was no statistical difference between each group at 1 day and 3 days, thus indicating similar proliferation capacity in each group at the early time. However, at 7 days, except for the control and RADA16 groups, every two groups showed significantly different proliferation capacities, and RADA16-CGFs had the best effect, while the control group had the worst (Figure 5c). Thus, RADA16 and CGFs could both accelerate cell proliferation, and when the two are used in combination, they synergistically promote cell proliferation.

### 3.5. Effect of RADA16-CGFs on Mineralization Capability

**Figure 6:**
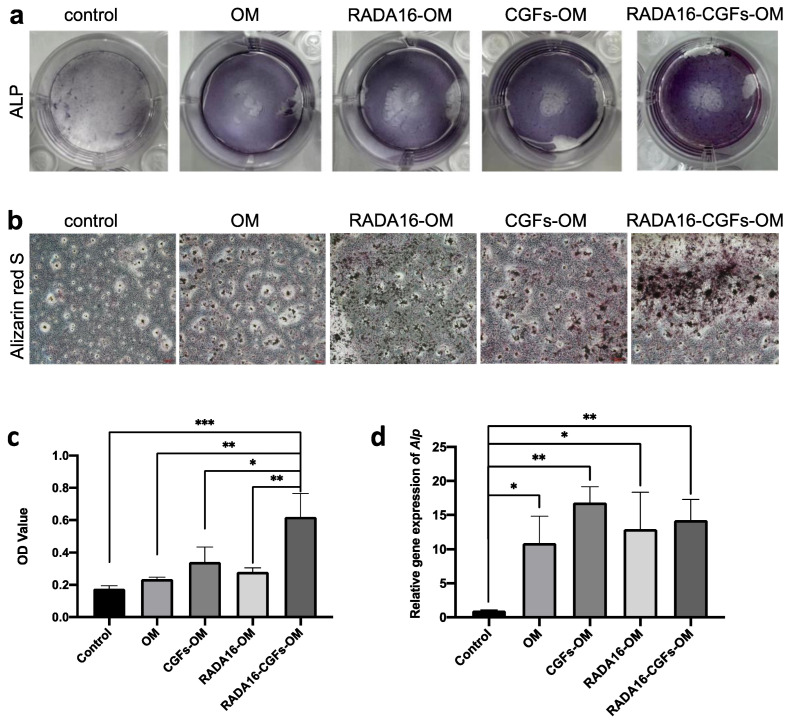
Effect of RADA16-CGFs on mineralization capability. (**a**) ALP staining images after culturing on the different composites for 7 days. (**b**) Optical microscopy images of alizarin red S staining after culturing on the different composites for 21 days. Scale bars, 100 μm. (**c**) Quantitative analysis of calcium nodules on the composites at a wavelength of 562 nm. *, *p* < 0.05; **, *p* < 0.005; ***, *p* < 0.001. (**d**) The gene expression of osteogenic differentiation-related proteins (*Alp*) of MC3T3 cells co-cultured with different composites. *, *p* < 0.05; **, *p* < 0.005.

Mineralization at 7 d in the different groups was confirmed by ALP staining. It demonstrated that the number of mineralized nodules in the RADA16-CGFs-OM group was the highest at 7 d, while the CGFs-OM group was more than the positive control, and the RADA16-OM group was similar to the positive control (Figure 6a). ARS staining was applied to further confirm the mineralization capability at 21d, which determined that CGFs alone could enhance the mineralization of MC3T3-E1 cells, but RADA16 alone might not have such enhancement (Figure 6b,c). RADA16-CGFs-OM, again, showed the best mineralization capability that the others had on MC3T3-E1 cells. The q-PCR of *Alp* again confirmed that CGFs-OM, RADA16-OM, and RADA16-CGFs-OM could promote mineralization capability (Figure 6d).

## 4. Discussion

Alveolar clefts are common congenital bony defects extending over the alveolar and process toward the hard palate and nasal cavity [28]. To reconstruct the maxillary arch, the autogenous bone from the iliac bone is one of the most common choices in the clinic, which can help induce the formation of new bone. However, the process is complex and risky and will cause trauma to the patient when collecting the bone [29]. The other choice is commercial bone grafting materials, such as Bio-OSS, which can serve as scaffolds for cell migration and vessel regeneration [30], but osteoinduction is not as good as autogenous bone.

To solve the aforementioned problem, blood-derived growth factors are explored and have already been used in the clinic [31], and CGFs are the most widely used concentrations. CGFs can release growth factors locally to enhance tissue regeneration, such as bone remodeling and wound healing [17,18], which are clinically applied to increase the success rate of bone grafting and implant treatment. However, the rapid release of growth factors from CGFs does not match the complex process of bone formation [27], so the potential of CGFs is not fully utilized, and the growth factors can be wasted after being released. Thus, to make the utmost of the CGFs, figuring out a maneuver to slow down and extend the release process should be the first choice. In this study, we collected all the samples for fabricating the CGFs from the same six healthy people who were 20–30 years old, as the CGFs of different people might have different effects.

RADA16, a widely used SAP that can form a stable nanofiber scaffold structure similar to the extracellular matrix (ECM), has been proven to be able to serve as a good environment for cell migration, proliferation, and differentiation [23], and can support the surrounding tissue regeneration [32]. Meanwhile, it can provide the possibility of slow and sustained release of growth factors due to its stable network structure. Thus, we hypothesized that the RADA16 nanofiber scaffold hydrogel could provide a suitable structure and environment for controlling the growth factors released from CGFs.

The SEM images of the RADA16 scaffold showed a stable and polyporous nanostructure.

We first tested whether the growth factors released from CGFs could be slowed down after being wrapped with RADA16 nanofiber scaffold hydrogel. VEGF can induce vessel regeneration, endothelial cell migration, and proliferation, which can help enhance osteogenesis during bone repairing [33]. PDGF can speed tissue recovery and induce osteoblasts and endothelial cell proliferation, which promote bone regeneration [34,35]. Therefore, we chose these two growth factors for measuring release speed. According to a publication [9,27], in pure CGFs, the speed of VEGF and PDGF release showed an increasing pattern from 1 d to 10 d. In our study, after being wrapped by RADA16, the RADA16-CGFs showed stable releasing patterns. For PDGF-BB, the releasing speed increased at 5 d and 7 d, but at 10 d, the speed again slowed down, which is different from the pure CGFs; for VEGF, the releasing speed was always the same from 1 h to 10 d. Thereafter, we confirmed the sustained release function of RADA16 application to CGFs. The assembly of RADA16 with a stable three-dimensional structure can lead to the slow and sustained release of growth factors from CGFs. RADA16 can form three-dimensional pores with similar diameters, providing an ideal network structure for a three-dimensional cell culture. At the same time, this pore-size regime provides the potential for the release of functional proteins [36,37,38].

The RADA16 nanofiber scaffold hydrogel can provide a suitable microenvironment for cell proliferation and functional protein action as it can highly simulate the ECM structure [37]. During the sustained release process, the bioactivity of CGFs can be maintained. In our study, the morphology of MC3T3-E1 cells was not affected by the application of CGFs and RADA16, in which there was no obvious change in morphology, cell apoptosis, or degenerescence, showing that RADA16 and CGFs were atoxic and safe for cells. The measurement of cell adhesion and CCK-8 testing further confirmed that CGFs, RADA16, and RADA16-CGFs enhanced cell proliferation. In the cell adhesion experiment, cells in the RADA16-CGF group started to be more than the control 60 min after cell seeding, and the CGF group performed the best at 10 min, which again demonstrated the sustained release property of RADA16. Meanwhile, the outcomes revealed that the RADA16 nanofiber scaffold hydrogel could serve better as a cultural environment by providing a 3D polyporous nanostructure [39]. CCK-8 testing also confirmed that there was no cytotoxicity of CGFs or RADA16, which was the same as the published results [40,41].

Finally, we assessed the effects of RADA16-CGFs on the mineralization capability of MC3T3-E1 cells. ALP staining was applied to assess the early stage (7 d) of the osteogenesis induction of RADA16-CGFs on MC3T3-E1 cells, while ARS staining was used to measure the mineralized nodule at 21 d. At both 7 d and 21 d, CGFs and RADA16 alone can help promote the mineralization of MC3T3-E1 cells, and RADA16-CGFs showed the most enhancement. The enhancement was further confirmed by q-PCR of ALP gene expression. Thus, we found that with the help of the RADA16 nanofiber scaffold hydrogel, the function of CGFs for promoting osteogenesis could be enhanced, which showed great potential for clinical application.

In conclusion, we provide a new method for endowing the sustained release of CGFs, which can help maximize the function of CGFs in osteoinduction. The application of the atoxic RADA16 nanofiber scaffold hydrogel with CGFs can be a new therapeutic strategy for the treatment of alveolar bone loss and other problems that require tissue regeneration. However, the limitation of our study is the lack of in vivo experiments, which should be carried out in the future.

## Data Availability

Raw data supporting the conclusion of this paper will be provided by the author without improper reservation.
